# Aging-Associated Amyloid-β Plaques and Neuroinflammation in Bottlenose Dolphins (*Tursiops truncatus*) and Novel Cognitive Health-Supporting Roles of Pentadecanoic Acid (C15:0)

**DOI:** 10.3390/ijms26083746

**Published:** 2025-04-16

**Authors:** Stephanie Venn-Watson, Eric D. Jensen

**Affiliations:** 1 Epitracker, Inc., San Diego, CA 92106, USA; 2 Seraphina Therapeutics, Inc., San Diego, CA 92106, USA; 3 U.S. Navy Marine Mammal Program, San Diego, CA 92152, USA

**Keywords:** Alzheimer’s disease, C15:0, pentadecanoic acid, dolphins, fatty acid amide hydrolase (FAAH) inhibitor, monoamine oxidase B (MAO-B) inhibitor, neuroinflammation, amyloid beta plaques, neuroprotectant, cognitive health

## Abstract

There is an urgent need to identify interventions that broadly target aging-related cognitive decline and progression to Alzheimer’s disease (AD). Bottlenose dolphins (*Tursiops truncatus*) have histologic changes similar to AD in humans, and they also develop shared age-associated co-morbidities identified as risk factors for AD in humans, including type 2 diabetes, ferroptosis, and iron overload, which can be driven by nutritional C15:0 deficiency. We hypothesized that (1) dolphins would have amyloid beta (Aβ) plaques and neuroinflammation that paralleled that of humans in relation to age-related progression, quantitative concentration, and brain region; and (2) C15:0 would have dose-dependent activities relevant to protecting cognitive health. Quantitative immunohistochemistry staining was used to assess 68 tissues from archived brains of 19 Navy dolphins to evaluate associations among amyloid beta (Aβ) plaques and neuroinflammation by brain region, sex, and age group. Further, dose-dependent C15:0 activities, using a third-party panel intended to screen for potential AD therapeutics, were evaluated. Similar to humans, dolphins had the highest Aβ plaque density variation in the hippocampus (90th percentile of 4.95 plaques/mm^2^), where plaque density increased with age (*p* = 0.05). All measured markers of neuroinflammation were detected, including the highest concentrations of activated microglia (CD68^+^) in the hippocampus (0.46 ± 0.38 cells/mm^2^). C15:0 was a dose-dependent inhibitor of two targets, fatty acid amide hydrolase (FAAH) (IC_50_ 2.5 µM, 89% maximum inhibition at 50 µM relative to URB597) and monoamine oxidase B (MAO-B) (IC_50_ 19.4 µM, 70% maximum inhibition at 50 µM relative to R(-)-Deprenyl). These activities have demonstrated efficacy against Aβ formation and neuroinflammation, including protection of cognitive function in the hippocampus. These findings suggest that, in addition to protecting against AD co-morbidities, C15:0 may play a distinct role in supporting cognitive health, especially at higher concentrations.

## 1. Introduction

Cognitive health declines with age, and this decline can progress to neurodegenerative disease. Alzheimer’s disease (AD) is the most common among neurodegenerative diseases, and the global prevalence of AD is expected to rise to 78 million people by 2030 [1]. While new drugs to modify AD have been approved by the Food and Drug Administration, there is an ongoing need to identify approaches that address early risk factors for age-related cognitive decline and AD [2].

It is now well understood that chronic co-morbidities of aging, including chronic inflammation, metabolic syndrome, iron overload, oxidative stress, type 2 diabetes, cardiovascular disease, and hearing loss, can increase the risk and severity of cognitive decline and neurodegenerative diseases, including Alzheimer’s disease (AD) [3,4,5,6,7,8,9,10]. One of the core limitations of animal models for AD is that these models are often limited to inducing the central nervous system components of AD (such as amyloid-β plaques, hyperphosphorylated tau proteins, and neurofibrillary tangles), but these are often absent of multiple naturally occurring chronic co-morbidities. As such, there is a meaningful gap in addressing early, common pathophysiological drivers among these co-occurring aging-related diseases.

The U.S. Navy Marine Mammal Program (MMP) has cared for a population of bottlenose dolphins housed in the open ocean for over 60 years. Due to the MMPs vigilant care of this population, Navy dolphins live longer than their counterparts in the wild; while the median lifespan of a wild dolphin is 20 years, more than one-third of the Navy’s current dolphin population is over 30 years old, with anticipated working lifespans to reach at least 50 years [11]. As dolphins age, they are susceptible to health changes similar to older humans, including chronic inflammation, metabolic syndrome, insulin resistance, fatty liver disease, hypercholesterolemia, and iron overload [12,13,14,15,16].

Fatty acid and broader metabolomic studies revealed that bottlenose dolphins with higher circulating concentrations of odd-chain saturated fatty acids (OCFAs C15:0 and C17:0) have a lower risk of having metabolic syndrome and related conditions; further, increasing dolphins’ dietary OCFAs results in improved cardiometabolic, iron, and red blood cell indices [17,18]. Since these initial discoveries, C15:0 has emerged as an essential fatty acid required by animals to support both early development and long-term health, especially related to metabolic, heart, liver, and red blood cell health [19,20,21,22,23]. Along with providing structural support for cell membranes, C15:0 is a pleiotropic nutrient with numerous mechanisms of action relevant to healthy aging, including its role as an AMPK, AKT, and dual PPARα/δ activator and an mTOR, JAK-STAT, and HDAC-6 inhibitor [19,23,24,25,26,27,28]. Further, C15:0 has broad anti-inflammatory, antifibrotic, antioxidant, anticancer, and antimicrobial activities [28,29,30,31,32,33]. In vivo studies with relevant animal models have shown that C15:0 effectively lowers cholesterol, triglycerides, glucose, and inflammation, and attenuates liver fibrosis, iron overload, anemia, tumors, and inflammatory bowel disease [19,31,34,35]. In controlled clinical trials, C15:0 supplementation raised circulating C15:0 levels, resulting in lowered LDL cholesterol, lower liver enzymes (ALT and AST), raised hemoglobin, and improved gut microbiome health [36,37]. While most C15:0 studies have focused on its role as an essential nutrient that protects metabolic, heart, and liver health, there are a few studies linking C15:0 to mental and cognitive health [30,38,39]. Specific to cognition, Shen et al. assessed potential correlations between plasma fatty acid levels and cognitive test scores among 372 adult patients (average age of 58 years, range of 52–63) with type 2 diabetes [38]. This study showed that people with higher C15:0 concentrations had higher total Mini-Mental State Examination (MMSE) scores, including higher MMSE orientation force scores. People with higher C15:0 concentrations also had higher total Montreal Cognitive Assessment (MoCA) scores as well as higher visual-spatial ability scores. Due to their demonstrated correlations between higher C15:0, improved delayed recall, and the quantitative insulin sensitivity check index (QUICKI), the authors attributed associations between higher C15:0 and better cognition to C15:0’s ability to improve insulin sensitivity.

Interestingly, the core histologic changes of AD in humans, including amyloid-β plaques, hyperphosphorylated tau proteins, and neurofibrillary tangles, have been well documented in the brains of numerous wild cetacean species, including bottlenose dolphins (*Tursiops truncatus*), striped dolphins (*Stenella coeruleoalba*), Atlantic spotted dolphins (*Stenella frontalis*), common dolphins (*Delphinus delphis*), Risso’s dolphins (*Grampus griseus*), white-beaked dolphins (*Lagenorhynchus albirostis*), Cuvier’s beaked whales (*Ziphius cavirostris*), and long-finned pilot whales (*Globicephala melas*) [40,41,42,43,44,45]. Further, based on assessments of lesions consistent with AD in the parietal lobe of wild cetaceans, there is evidence that these neurodegenerative changes increase with age [45]. Among all animals, only primates and cetaceans naturally develop histologic changes that include amyloid-β plaques, hyperphosphorylated tau proteins, and neurofibrillary tangles, and as such, it is hypothesized that these changes are a result of longevity and post-reproductive lifespan, not just aging [40,46].

Due to: (1) C15:0’s role as an essential fatty acid with robust evidence of broad benefits that protect against multiple co-morbidities of aging closely linked to AD, including chronic inflammation, metabolic syndrome, and iron overload in both humans and Navy dolphins; (2) associations found between higher plasma C15:0 concentrations and better cognitive scores among people with type 2 diabetes; and (3) the known presence of histologic neurodegenerative changes consistent with AD in wild dolphins, we hypothesized that (1) older Navy dolphins would have amyloid beta (Aβ) plaques and neuroinflammation that paralleled that of humans in relation to age-related progression, quantitative concentration, and brain region; and (2) C15:0 would have dose-dependent activities relevant to protecting cognitive health. The current study’s confirmation of aging-associated brain changes in the well-studied Navy dolphin population, which also has aging and AD-related co-morbidities that are effectively attenuated by raising dietary C15:0 intake, may also open avenues into potential protective factors for AD. The discovery of C15:0 as both a fatty acid amide hydrolase (FAAH) and monoamine oxidase B (MAO-B) inhibitor, especially at higher concentrations, furthers the potential importance of C15:0 to support cognitive health in long-lived, large-brained mammals. Additional studies, including clinical trials, are needed to determine whether C15:0 plays a direct role in preventing or slowing the onset and progression of AD.

## 2. Results

### 2.1. Evaluation of Aβ Plaques and Neuroinflammation in Dolphins

#### 2.1.1. The Hippocampus Had the Greatest Variability of Aβ Plaque Density, and Older Dolphins Had the Highest Aβ Plaque Density

**Figure 1:**
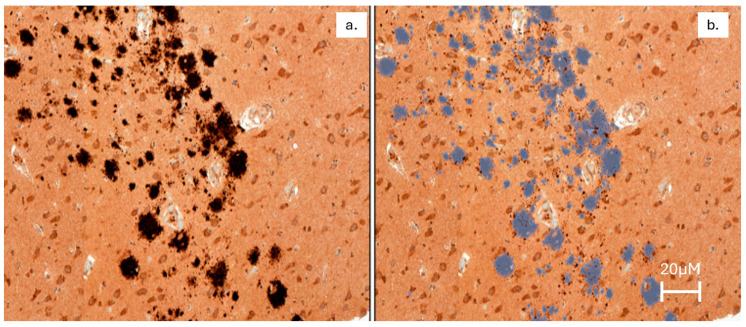
The original image (**a**) and the use of masking (**b**) used to identify amyloid beta (Aβ) plaques in the hippocampus of a bottlenose dolphin (*Tursiops truncatus*), represented in gray.

Of 68 brain tissues assessed, at least one Aβ plaque was detected in 63 (93%) samples representing all 19 dolphins (Figure 1). Aβ plaque density in the hippocampus ranged from 0 to 46 plaques per mm^2^ with quantile distribution as follows: 10% = 0.00, 25% = 0.04, 50% = 0.61, 75% = 0.90, 90% = 4.95. The greatest variation in Aβ plaque density was in the hippocampus (Table 1). There were no significant differences in any of the Aβ plaque measurements when comparing by sex (*p* < 0.05).

**Figure 2:**
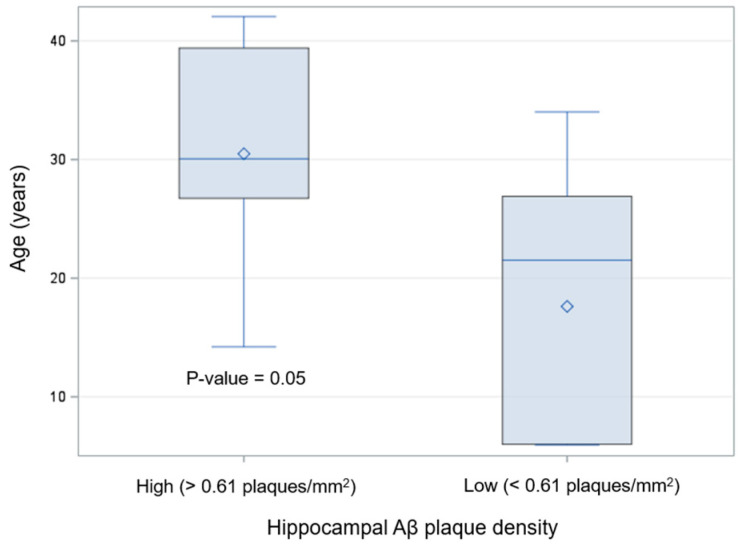
Box plot of measured Aβ plaque densities in the hippocampus of bottlenose dolphins (*Tursiops truncatus*) (*n* = 19) using quantitative immunohistochemical (IHC) staining. The top and bottom bars at the end of the whiskers represent the minimum and maximum data points, respectively, as well as the measurement range; the top and bottom of the box represent the upper quartile Q3 and lower quartile Q1, respectively; the middle bar in the box is the median; the whole box represents the interquartile range; and the diamond represents the mean. The *p*-value = 0.05 is based on a Kruskal–Wallis test comparing dolphins’ age by high or low Aβ plaque density (defined as > or <0.61 plaques/mm^2^, respectively).

Dolphins with higher Aβ plaque density (more than 0.61 plaques/mm^2^) were older than dolphins with lower Aβ plaque density (less than 0.61 plaques/mm^2^) (30 ± 11 versus 18 ± 11 years old, respectively; *p* = 0.053) (Figure 2). There were no significant differences in mean Aβ staining indices across the four tissue types, with the exception of mean Aβ plaque staining, which was lowest in the frontal cortex and highest in the red nucleus (Table 1).

#### 2.1.2. Neuroinflammation Was Present in Dolphin Brains, with the Greatest Microglial Activation in the Hippocampus of Dolphins

**Figure 3:**
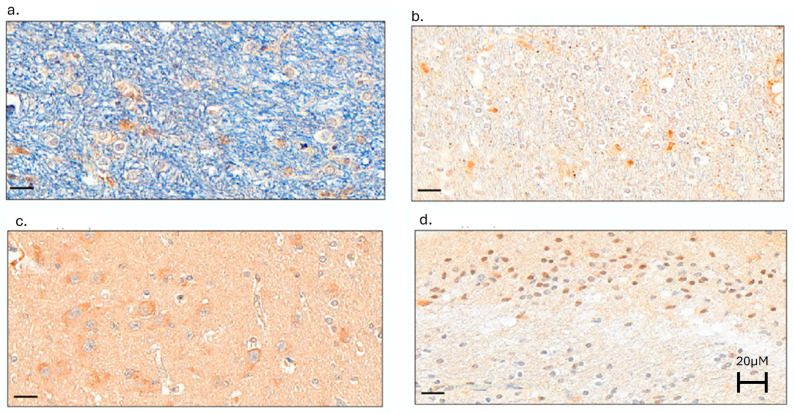
Representative images of IHC staining on FFPE bottlenose dolphin (*Tursiops truncatus*) brain samples, including positive immunoreactivity visualized with DAB (brown) for (**a**) IL-6 in the red nucleus, (**b**) IFN-γ in the hippocampus, (**c**) TNF-α in the hippocampus, and (**d**) TGF-β1 in the hippocampus.

All indices of neuroinflammation were detected in dolphin brains (Figure 3).

There were no differences in IHC staining across the four tissue types, with the exception of the highest CD68+ cell concentrations (microglia activation) in the hippocampus and the lowest concentrations in the substantia nigra (Table 2). Compared to males, females had higher mean IL-6 (3.8 ± 2.2 and 5.2 ± 2.7) and TGF-β (6.0 ± 2.7 and 7.1 ± 2.9) staining. Dolphins with lower IFN-γ staining intensity (less than 5.1) were older than dolphins with higher IFN-γ staining intensity (more than 5.09) (26 ± 10 versus 18 ± 14 years old, respectively; *p* = 0.017). No other IHC stains differed significantly by age.There were differences in mean IHC stain intensity by sex for IL-6 and TGF-β (*p* = 0.015 and 0.039, respectively).

### 2.2. C15:0 Neuroprotective Mechanisms

Of the 39 assays described in the methods section of this study, C15:0 had two had significant inhibitory activities: fatty acid amide hydrolase (FAAH) and monoamine oxidase MAO-B (Table 3, Supplementary Table S1).

#### 2.2.1. Pentadecanoic Acid (C15:0) Is a Fatty Acid Amide Hydrolase (FAAH) Inhibitor

**Figure 4:**
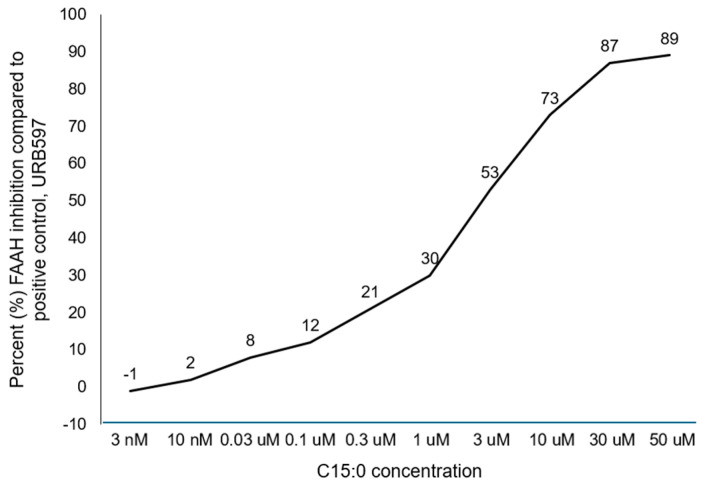
Response curve of C15:0 inhibition activities, based on the average of duplicate runs at each concentration, against fatty acid amide hydrolase (FAAH) compared to the positive control (URB597). Data as reported by Eurofins Discovery as part of its Alzheimer’s MoA LeadHunter Panel using standardized enzyme and radioligand binding assays. These data are based on 10 ascending concentrations (3 nM to 50 µM) of pure, free fatty acid C15:0.

Pure C15:0 is a significant inhibitor of fatty acid amide hydrolase (FAAH), with an IC_50_ of 2.51 µM. C15:0’s maximum effective percent inhibition compared to the positive control (URB597) was 89% at 50 µM. The dose-response curve of these inhibition activities is shown in Figure 4.

#### 2.2.2. Pentadecanoic Acid (C15:0) Is a Monoamine Oxidase (MAO-B) Specific Inhibitor

**Figure 5:**
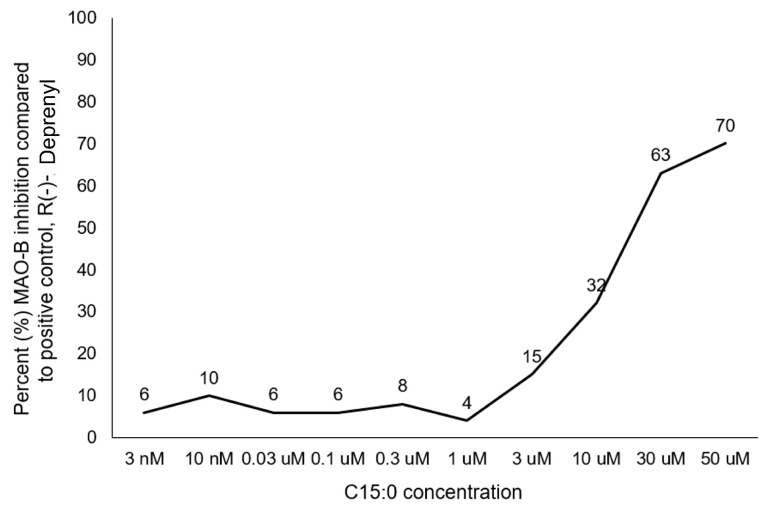
Response curve of C15:0 inhibition activities, based on the average of duplicate runs at each concentration, against monoamine oxidase MAO-B compared to the positive control (R(-)-Deprenyl). Data as reported by Eurofins Discovery as part of its Alzheimer’s MoA LeadHunter Panel using standardized enzyme and radioligand binding assays. These data are based on 10 ascending concentrations (3 nM to 50 µM) of pure, free fatty acid C15:0.

Pure C15:0 is a significant inhibitor of monoamine oxidase MAO-B, with an IC_50_ of 19.4 µM. C15:0’s maximum effective percent inhibition compared to the positive control (R(-)-Deprenyl) was 70% at 50 µM. The dose-response curve of these inhibition activities is shown in Figure 5.

## 3. Discussion

This study provides a novel assessment of brain changes specific to Aβ plaques and neuroinflammation, by brain region, in bottlenose dolphins. Here, we show that the highest variation of Aβ plaques, along with the highest density of activated microglia, was present in the hippocampus. Further, hippocampal concentrations of Aβ plaques increased with age. These histological findings are consistent with those present in humans with AD [47]. Further, this study reported average Aβ plaque densities within the dolphin hippocampus (3.8 plaques per mm^2^) that lie between those reported in elderly humans with no clinical signs of dementia (0.2 ± 0.1 plaques/mm^2^) and cases with severe disorientation and memory impairment, but no aphasia, apraxia, and agnosia (5.7 ± 1.1 plaques/mm^2^) [48]. The hippocampus is primarily responsible for learning and long-term memory (recalling events from the past), and it also has a role in future planning and decision-making, which explains why memory loss and learning difficulties are core components of AD [47,49]. Inflammatory microglia are particularly active in the hippocampus, and neuroinflammation plays an important role in cognitive impairment and dementias, including AD [47,50]. Whether these age-related histological changes shared between dolphins and humans translate to similar behavioral changes in dolphins is yet to be determined.

This and other studies continue to demonstrate strong parallels between histologic changes consistent with AD in humans and cetaceans, including dolphins [40,41,42,43,44,45]. While many genetic and environmental differences in human populations have made understanding the drivers of the underlying pathophysiology of AD difficult, bottlenose dolphins have much less complex and more consistent lifestyles, including fish-based diets, constant physical activity, socialization throughout their lives, and (for Navy dolphins) equal access to routine health care [51]. As a result, the care of Navy dolphins has helped to elucidate a single pathophysiology of chronic co-morbidities of aging, including anemia, dysmetabolic iron overload syndrome (DIOS), metabolic dysfunction-associated steatotic liver disease and steatohepatitis (MASLD and MASH), and insulin resistance [52]. In dolphins, a discovered core underlying driver is nutritional C15:0 deficiency and its clinical outcome, Cellular Fragility Syndrome.

Specifically, low dietary C15:0 intake results in low erythrocyte membrane C15:0 concentrations (<5 µg/mL), which increases the fragility of red blood cells [52]. These fragile red blood cells are engulfed by liver macrophages (Kupffer cells), which, over time, cause liver iron overload. Fragile cell membranes that are susceptible to increased lipid peroxidation, paired with iron overload, culminate in ferroptosis, a newly discovered form of cell death that causes massive reactive oxygen species (ROS) production and deactivated mitochondria [53]. In turn, ferroptosis spills over from the liver to seed other organs, including the pancreas, heart, and brain. Systemic ferroptosis accelerates the onset and progression of aging-associated co-morbidities, including anemia, DIOS, MASLD, MASH, type 2 diabetes, heart disease, and neurodegeneration [52,53].

Specific to AD, there is increasing evidence that ferroptosis plays a role in inciting and advancing AD, and targets for ferroptosis have been proposed as potential therapeutics for AD [54,55]. Post-mortem evaluations of brain tissues from people with AD demonstrated that the progression of AD was accompanied by key components of the ferroptosis pathway. Further, in human organoid AD systems, prevention of ferroptosis prevented Aβ pathology, lowered lipid peroxidation, and restored appropriate iron handling by the tissues [56]. The emerging pathophysiology in humans linking ferroptosis to AD is as follows: ferroptosis and iron overload in the brain cause neuronal degeneration, which directly results in neurofibrillary tangles and, independently, Aβ plaque formation [57]. Free redox-active iron, present with ferroptosis, has been proposed as the core of AD in humans, opening up new and promising avenues to AD therapies [57].

In vitro and in vivo studies, as well as controlled clinical trials, have shown that C15:0 supplementation can raise circulating C15:0 concentrations above 5 µg/mL (20 µM), which attenuates or resolves all components of Cellular Fragility Syndrome, ferroptosis, and downstream components of co-morbidities of aging, including anemia, DIOS, MASH, type 2 diabetes, and hypercholesterolemia and hypertriglyceridemia, within 12 weeks [17,19,36,37,52]. Given this unique single syndrome in dolphins and the well-documented links between these co-morbidities of aging, including AD in humans, there is a need to understand the potential role of C15:0 deficiencies and Cellular Fragility Syndrome in the onset or progression of ferroptosis-driven AD in humans.

In addition to the promising use of C15:0 to prevent ferroptosis and downstream AD pathology, in the current study, C15:0 had two dose-dependent inhibitory activities that were detected using the Eurofins Discovery Alzheimer’s LeadHunter MoA Panel: inhibition of fatty acid amide hydrolase (FAAH) and monoamine oxidase B (MAO-B). C15:0 had maximum FAAH inhibition activities at 50 µM, which was 89% compared to the positive control (URB597). This optimal concentration of 50 µM is higher than concentrations previously reported for other C15:0 activities (i.e., 20 µM) to lower multiple proinflammatory markers and repair mitochondrial function at [19,26,52].

FAAH is a circulating enzyme that rapidly degrades anandamide, which is an endocannabinoid [58]. Endocannabinoids are endogenously produced molecules that activate cannabinoid receptors 1 and/or 2 (CB1 and CB2), which are present throughout the brain and body. The endocannabinoid system (ECS, including endocannabinoids and their receptors) is core to maintaining whole-body and brain homeostasis, including tempering overactive immune responses, managing pain, supporting healthy sleep, and calming mood [59]. The ECS declines in function with age, contributing to increased inflammation, poor sleep, and increased anxiety. Further, the ECS is neuroprotective, and declines in the ECS with age can also impair cognitive function. As such, compounds targeting the ECS, including FAAH inhibitors, have broad therapeutic potential [58,59]. Specific to AD, FAAH inhibitors have demonstrated protective effects against amyloidosis, resulting in improved cognitive function, improved memory, lower oxidative stress, and lower neuroinflammation in an AD mouse model [60]. FAAH inhibition results in lower neuroinflammation via autophagy recovery and greater neuroprotection in AD models [61,62]. Combination therapies using both FAAH-inhibitors and PPAR activators have been proposed as novel treatments for AD due to their abilities to modulate neuroinflammation and inhibit Aβ accumulation [63]. Further, because FAAH inhibitors have been demonstrated to lower neuroinflammation, lower pain, improve sleep patterns, and improve memory and locomotory activity, compounds that have this MoA have been proposed to help attenuate symptoms of AD [64].

The demonstrated role of C15:0 as both an FAAH inhibitor and PPARα/δ activator supports its potential added role of protecting cognitive health, including activities that can lower neuroinflammation and Aβ accumulation. This role is further strengthened by previous demonstration of C15:0 as a means to prevent and treat ferroptosis and tissue iron deposition; its demonstrated activities as an antioxidant, anti-inflammatory, and pro-mitochondrial activities, as well as being an mTOR inhibitor (which supports autophagy) and AKT activator, all of which have been shown to support cognitive health and proposed as potential approaches to treating AD in humans [2,50,65,66,67,68,69].

Beyond being protective against neuroinflammation and Aβ accumulation, FAAH inhibitors have been proposed as novel anxiolytic drugs [70]. These molecules have reversed early life stress (ELS)-induced depressive-like behaviors in relevant models by decreasing neuroinflammatory cytokines [71]. Social stress-induced depressive-like symptoms have been prevented by inhibiting FAAH in a relevant animal model [72]. Additionally, a clinical trial showed that FAAH inhibitors were effective anxiolytics for people with social anxiety disorder [73]. Interestingly, previously reported human cell phenotypic screening of C15:0 also showed significant parallels in anti-inflammatory activities between C15:0 and bupropion, a leading antidepressant [30]. Further, C15:0 has a metabolite, called pentadecanoylcarnitine (PDC), which is itself a fully acting endocannabinoid. Together, these data strengthen C15:0’s role in supporting mental health, including multiple roles that support the endocannabinoid system [74].

FAAH inhibitors may help treat chronic pain and alleviated stress-related visceral hypersensitivity (pain) in relevant models [75,76]. Additionally, FAAH inhibitors were protective to kidneys, specifically related to cisplatin-induced nephrotoxicity [77]. These studies further support that, given the broad benefits of FAAH inhibitors, C15:0’s health benefits may now include supporting cognitive health, mood, and alleviating pain.

The current study also identified C15:0 as a dose-dependent monoamine oxidase B (MAO-B) inhibitor. MAO-B inhibitors inhibit the reuptake of dopamine and sensitize dopaminergic neurons to physiological and pharmacological influences [78,79]. By doing so, MAO-B inhibitors help to keep higher dopamine levels in the brain. MAO-B inhibitors have been shown to be a useful adjunct to other therapeutics (i.e., levodopa) for AD and Parkinson’s disease (PD) with a high safety margin [79,80]. Specific to Aβ, evaluation of human brain tissues supports that rises in MAO-B precede the development of Aβ, and MAO-B appears to regulate the production of Aβ [81]. In mouse models, an MAO-B inhibitor significantly reduced both cerebral iron and Aβ plaque accumulation [82]. Further, MAO-B inhibitors have been shown to rapidly increase nitric oxide (NO) in both the brain tissue and cerebral vessels, with demonstrated protection of the vascular endothelium from Aβ-related damage [83]. As such, MAO-B inhibitors have been proposed as neuroprotectants [80,81,82,83].

MAO-B inhibitors have also been protective against AD-induced anxiety-like behavior and memory impairment in animal models, and they may have broader indications as a neuroprotectant across multiple neurodegenerative diseases [84,85,86]. On their own, MAO-B inhibitors can slow the rate of PD symptom progression and extend survival via neuroprotection and/or symptom relief; MAO-B inhibitors have been shown to ameliorate motor symptoms and improve motor fluctuation in both early and advanced stages of PD, and they may also help attenuate mood deflection, cognitive impairment, sleep disturbances, and fatigue [87,88].

Beyond their role in slowing the progression of neurodegenerative diseases by slowing the breakdown of dopamine, MAO-B inhibitors also lower oxidative stress and stimulate the production and release of neurotrophic factors (glial cell line-derived neurotrophic factor) that support dopaminergic neurons [89,90]. Additional demonstrated neuroprotective properties of MAO-B inhibitors include counteracting excessive glutamate and combating the negative effects of free radicals and exogenous neurotoxins in animal models [88,90]. Further, MAO-B inhibitors have anti-depressant activities and have shown efficacy in treatment-resistant depression [91].

In addition, MAO-B inhibitors have anti-aging properties that enhance and extend longevity. Dopamine levels decrease with age, which results in “normal” aging-associated cognitive impairment, as well as accelerated biological aging and shorter lifespans [92,93]. As such, by sustaining higher dopamine levels, MAO-B inhibitors may be used to enhance cognitive function with age. MAO-B inhibitors have been shown to extend lifespan in four different rat studies and one dog study [94,95]. Interestingly, MAO-B inhibitors have enabled rats to live beyond their known maximum lifespan [95]. Due to the many age-related benefits above, routine use of MAO-B inhibitors in humans, starting at the age of 45 years old, has been suggested to “delay the time of natural death and decrease the susceptibility of age-related neurological diseases” [96].

In the current study, C15:0 had maximum MAO-B inhibition at 50 µM, which was 70% compared to the positive control, R(-)-Deprenyl. Similar to C15:0’s FAAH inhibition activities, the optimal MAO-B inhibiting concentration was higher than C15:0 activities most relevant to metabolism, multiple proinflammatory markers, and mitochondrial function, which are optimal at 20 µM [19,27].

While a key limitation of this study is the small sample size (*n* = 19), the extent of analyses by brain section makes this one of the most extensive evaluations of Aβ plaques and individual inflammatory biomarkers, by brain region, to date. Another limitation includes the duration of storage of whole dolphin brains, which could impact the quality and accuracy of quantitative IHC staining. Given that our findings of increased Aꞵ plaques with age in the hippocampus of dolphins matched that found in human studies, support that at least the Aꞵ IHC readings were not impacted by storage duration. Regarding inflammation biomarkers, the expectation would be that cytokines degrade over time. As such, the presence of these cytokines in dolphin brain tissues still supports the presence of neuroinflammation, but the quantification trends of these biomarkers may be more limited due to storage time. For the targeted C15:0 mechanism-of-action assays, each of the ten concentrations was conducted in duplicate, and only the average was reported by Eurofins. Since data from each individual run by concentration were not reported, these data may be limited without an understanding of the variation between the two runs. The expected clean ascending trend in inhibition activities by increasing dose, however, increases the confidence that the applied averages are accurate representations of C15:0 activities.

In conclusion, this study showed that some older dolphins develop age-related changes in the brain, including Aβ plaques and neuroinflammation, which are core contributors to impaired cognition and AD in humans. These findings are aligned with previously established parallels between dolphins and humans related to chronic aging-associated conditions, including insulin resistance, dysmetabolic iron overload syndrome, metabolic syndrome, and chronic inflammation, all of which have been linked to an increased risk of ferroptosis and AD. In addition to C15:0’s demonstrated ability to reverse all components of ferroptosis, a promising neuroprotective target to prevent and treat AD, this study also showed that C15:0 has dose-dependent FAAH and MAO-B inhibition activities, both of which have neuroprotective and cognitive health-supporting mechanisms that also have the potential to prevent and attenuate AD in humans. Further studies are needed to assess C15:0’s potential direct role in stemming the rise in AD.

## 4. Materials and Methods

### 4.1. Experimental Design

Due to: (1) C15:0’s role as an essential fatty acid with robust evidence of broad benefits that protect against multiple co-morbidities of aging closely linked to AD, including chronic inflammation, metabolic syndrome, and iron overload in both humans and Navy dolphins; (2) associations found between higher plasma C15:0 concentrations and better cognitive scores among people with type 2 diabetes; and (3) the known presence of histologic neurodegenerative changes consistent with AD in wild dolphins, this study sought to: (1) evaluate archived Navy dolphin brains for histological age-associated changes including relative numbers of amyloid beta (Aβ) plaques and biomarkers of neuroinflammation by brain region, sex, and age group, and (2) test C15:0 for 39 dose-dependent mechanism of action (MoA)-based activities using the Alzheimer’s MoA LeadHunter Panel (Eurofins Discovery, Taipei, Taiwan), an independent, third-party run platform.

### 4.2. Study Population

This study assessed all available, whole, and intact archived formalin-fixed brains that had been stored for up to 10 years from bottlenose dolphins (*Tursiops truncatus*) previously cared for by the U.S. Navy Marine Mammal Program (*n* = 20). One dolphin was excluded from the study, as the tissue samples had extreme outlying staining values across multiple stains. Of the remaining 19 dolphins in the study, 7 (37%) were female, and 12 (63%) were male. The age range of dolphins in this study was 2 to 50 years old.

### 4.3. Histology

#### 4.3.1. Study Samples

Brains were provided to a pathology service (Reveal Biosciences, San Diego, CA, USA) in 10% neutral buffered formalin as whole, pre-sliced, or in parts. Each brain was sectioned into 0.5 cm-thick coronal slices for a total of 13 slices, each slice corresponding to a different region of interest. Each slice was then processed and embedded following the pathology services’ standard protocol. A total of 240 formalin-fixed paraffin-embedded (FFPE) blocks were generated. While most tissues sought in the dolphins were available, not every dolphin had all samples available for every desired tissue. A total of 68 FFPE tissues, frontal cortex (*n* = 18), hippocampus (*n* = 15), red nucleus (*n* = 18), and substantia nigra (*n* = 17) from each dolphin were sectioned at 4 µM. These slides were prepared for individual staining. 

#### 4.3.2. Immunohistochemical (IHC) Staining

IHC staining for amyloid beta (Aβ), interleukin-6 (IL-6), interferon-gamma (IFN-γ), tumor necrosis factor-alpha (TNF-α), transforming growth factor-beta 1 (TGF-β1), and cluster of differentiation 68 (CD68) was performed for each targeted and available tissue. A summary of antibodies used is provided in Table 4. When more than one species of host was available for any given positive control or antibody, the species most relevant to bottlenose dolphins, whether by pathophysiology of chronic age-associated diseases or by evolution, was selected.

Amyloid Beta (Aβ) Plaque Staining. IHC optimization was performed on formalin-fixed paraffin-embedded (FFPE) dolphin sample 17-002 MAK B05, using a Leica Bond automated immunostainer. Amyloid beta (Aβ) was tested at four different dilutions plus a negative control (no primary antibody): 1:100, 1:200, 1:400, and 1:800. Heat-induced antigen retrieval was performed using Leica Bond Epitope Retrieval Buffer 1 (Citrate solution, pH6.0) for 20 min (ER1(20)) or Leica Bond Epitope Retrieval Buffer 2 (EDTA solution, pH9.0) for 20 min (ER2(20)). Non-specific background was blocked with 5% milk in PBS-T. Beta-Amyloid antibody was detected using Novocastra Bond Refine Polymer Detection and visualized with 3′3-diaminobenzidine (DAB; brown). A Hematoxylin nuclear counterstain (blue) was applied, and optimization slides were examined. It was determined that 1:200 with ER2(20) provided the optimal staining conditions. Aβ IHC was performed on one slide per sample from the cortex (frontal), hippocampus, substantia nigra, and red nucleus blocks. FFPE human midbrain was used as a positive control. A negative control (no primary) was performed on dolphin brain tissue.

Neuroinflammation Biomarkers. The following cytokines and inflammatory markers were detected and measured using quantitative IHC: interleukin-6 (IL-6) and interferon-gamma (IFN-γ). tissue necrosis factor-alpha (TNF-α), transforming growth factor-beta (TGF-β), and cluster of differentiation 68 (CD68). Briefly, immunohistochemistry (IHC) optimization was performed on FFPE sample 17-002 MAK B05, using a Leica Bond automated immunostainer. Inflammatory marker antibodies were tested at four different dilutions, plus a negative control with no primary antibody: 1:100, 1:200, 1:400, and 1:800. Heat-induced antigen retrieval was performed using Leica Bond Epitope Retrieval Buffer 1 (Citrate solution, pH pH6.0) for 20 min (ER1(20)) or Leica Bond Epitope Retrieval Buffer 2 (EDTA solution, pH pH9.0) for 20 min (ER2(20)). Non-specific background was blocked with 5% milk in PBS-T. Inflammatory biomarker antibodies were detected using Novocastra Bond Refine Polymer Detection and visualized with 3′3-diaminobenzidine (DAB; brown), and a hematoxylin nuclear counterstain (blue) was applied. Optimization slides were examined, and the following optimal staining conditions were determined for each neuroinflammation biomarker: 1:100 with ER1(20) for IL-6, TNFα, and CD68; 1:1600 with ER1(20) for IFN-γ; 1:250 with ER2(20) for TGF-β. IHC was performed on one slide per sample from the cortex (frontal), hippocampus, substantia nigra, and red nucleus blocks. FFPE The following tissues were used as positive controls for FFPE: human lung for IL-6, human midbrain for IFN-γ, human tonsil for TNFα and CD68, and mouse gastrointestinal tissue for TGF-β. A negative control (no primary) was performed on dolphin brain tissue. Available positive control animals and tissues for each inflammation biomarker were limited, and species with brains most closely aligned with dolphins (i.e., humans, ideally from brain tissues) were prioritized. If human tissue positive controls were not available, then mouse positive controls were used.

#### 4.3.3. Whole Slide Imaging

Whole slide images were generated in bright field using a Pannoramic SCAN (3D Histech, Budapest, Hungary) and are provided on a USB data drive together with image viewing software. Quality Control: Images were assessed for quality by an image specialist, and any images not meeting our quality criteria were rescanned. Representative snapshot images are included in this report. Magnification for all images is 40×. The scale bar represents 20 µm.

#### 4.3.4. Digital Image Analysis

Image analysis data were generated by automated analysis of whole slide images using ImageDx™ Software Version 2.1. This software integrates tissue section and color deconvolution-based quantification to organize and characterize data for each slide. The color deconvolution quantification algorithm first isolated marker-positive image data from the total tissue image data. The algorithm identified tissue with respect to location on the slide and then the positively stained regions for Turnbull’s or Prussian blue staining. These identified regions were then quantified for precise positivity, corresponding to neurons.

#### 4.3.5. Data Analysis

SAS Statistical Software Version 9.2 (SAS Institute, Cary, NC, USA) was used to compare quantitative IHC staining data by brain section (frontal cortex, hippocampus, red nucleus, and substantia nigra), age category (younger = less than 25 years old, older = greater than or equal to 25 years old), and sex using Wilcoxon rank sums (for brain sections) and Kruskal–Wallis tests (for age categories and sex). These non-parametric statistical methods were chosen due to the small sample size and non-normal distribution of the data. Wilcoxon rank sums were used to compare multiple variables (brain sections), and Kruskal-Wallis tests were used to compare 2-category independent variables with linear dependent variables. Significance was defined as a *p* value ≤ 0.05, and the data were presented as mean ± SD to enable the reader to see both the mean and the data variability.

### 4.4. C15:0 Mechanistic Activities Relevant to Cognitive Health

Standardized enzyme and radioligand binding assays were used by an independent third party (Eurofins Discovery, Taiwan) to evaluate C15:0 across ten concentrations and up to 50 µM for activities as part of the Eurofins Discovery Alzheimer’s MoA LeadHunter Panel. Methods employed in this study were adapted from the scientific literature to maximize reliability and reproducibility. Reference standards were run as an integral part of each assay to ensure the validity of the results obtained. IC50 values were determined by a non-linear, least squares regression analysis using MathIQTM (ID Business Solutions Ltd., Woking, UK).

This study included evaluated inhibition of the following targets by C15:0 using enzyme assays (Cholinesterase, Acetyl, ACES; Cholinesterase, Butyryl, CHLE; Peptidase, CTSB (Cathepsin B); Cyclooxygenase COX-1; Cyclooxygenase COX-2; Myeloperoxidase; IDO1; Monoamine Oxidase MAO-A; Monoamine Oxidase MAO-B; Peptidase, Endothelin Converting Enzyme-1 (ECE-1); Peptidase, Metalloproteinase, Neutral Endopeptidase; Peptidase, BACE1 (beta-Secretase); Protein Tyrosine Kinase, Fyn; Protein Serine/Threonine Kinase, GSK3B; Protein Serine/Threonine Kinase, MAPK14 (p38alpha); Protein Tyrosine Phosphatase, DUSP22; Fatty Acid Amide Hydrolase (FAAH); Nitric Oxide Synthetase, Inducible (iNOS); Catechol-O-Methyl Transferase (COMT); and Lipoxygenase 15-LOX-2) and radioligand binding assays (Adenosine A2A; Transporter, GABA; GABAA, Ro-15-1788, Hippocampus; Glutamate, AMPA; Glutamate, NMDA, MK-801; Glutamate, NMDA, Polyamine; Glutamate, Metabotropic, mGlu5; Histamine H3; Chemokine CX3CR1; Muscarinic M1; Nicotinic Acetylcholine alpha7, Methyllycaconitine; Serotonin (5-Hydroxytryptamine) 5-HT1A, WAY-100635; Serotonin (5-Hydroxytryptamine) 5-HT4B; Serotonin (5-Hydroxytryptamine) 5-HT6; Sodium Channel, Site 1; Somatostatin sst4; Sigma 2; Sigma1; Platelet Activating Factor (PAF)).

Biochemical assay results were presented as the percent inhibition of specific binding or activity. In addition to reporting the lowest concentration with a significant response judged by the assays’ criteria, where applicable, either the secondary assay results with the lowest dose/concentration meeting the significance criteria or, if inactive, the highest dose/concentration that did not meet the significance criteria were reported. Primary screening was conducted in duplicate with semi-quantitative data (e.g., estimated IC50). Significant responses (≥50% inhibition) were noted. Specific methodologies for assays in which positive results were reported are provided below.

#### 4.4.1. Fatty Acid Amide Hydrolase (FAAH) Inhibition

Using standardized methods previously described [82], this enzyme assay used human recombinant Sf21 cells with a 20 µM AMC arachidonolyl amide substrate and 1.0% DMSO vehicle. The time and temperature during pre-incubation were 15 min at 37 °C, and during incubation were 60 min at 37 °C. The incubation buffer was 125 mM Tris-HCl, pH of 9.0, and 1 mM EDTA/NaOH. The method of quantitation was spectrofluorimetric quantitation of 7-amino-4-methylcoumarin. The significance criterion was ≥50% of maximum inhibition compared to the reference positive control (URB597).

#### 4.4.2. Monoamine Oxidase B (MAO-B) Inhibition

Using standardized methods [83,84], this enzyme assay used human recombinant insect cells with a 50 µM kynuramine substrate and 1.0% DMSO vehicle. The time and temperature during pre-incubation were 15 min at 37 °C, and during incubation were 60 min at 37 °C. The incubation buffer was 100 mM potassium phosphate, pH 7.4. The method of quantitation was spectrofluorimetric quantitation of 4-hydroxyquinolone. The significance criterion was ≥50% of maximum inhibition compared to the positive control (R(-)-Deprenyl).

## 5. Conclusions

In conclusion, vigilant long-term care of U.S. Navy dolphins continues to provide opportunistic insights into the natural pathophysiology of co-morbidities of aging in long-lived mammals, including anemia, iron overload, ferroptosis, fatty liver disease, heart disease, and type 2 diabetes, all of which have been linked to fixable nutritional C15:0 deficiencies in dolphins and humans. As an extension of this research, the current study showed that Navy dolphins, similar to humans and wild dolphins, can also develop histologic changes in the brain consistent with AD, including neuroinflammation and Aβ plaques in the brain that increase in the hippocampus with advanced age. Just as importantly, this study showed that C15:0 has newly discovered, direct dose-dependent neuroprotective activities relevant to addressing AD and aging-related cognitive changes. As an FAAH and MAO-B inhibitor, C15:0 has optimal activities at 50 µM that can protect cognitive health, especially with age. These latest discoveries are particularly important given the growing understanding of C15:0 to support human longevity.

## 6. Patents

U.S. Provisional Patent 63/753836.

## Figures and Tables

**Table 1:** Comparisons of bottlenose dolphin (*Tursiops truncatus*) brain immunohistochemical amyloid beta (Aβ) staining across four sections (*n* = 19 dolphins). Wilcoxon rank sums test was used for comparisons among brain sections. Significance was defined as a *p* value ≤ 0.05.

Aβ Staining by Brain Section	Aβ Plaque Density (Plaques/mm^2^)	Aβ Plaques by Area (%)	Aβ Plaque Count	Mean Aβ Plaque Staining	Total Area of Aβ Plaques (mm^2^)
	Mean ± SD	Mean ± SD	Mean ± SD	Mean ± SD	Mean ± SD
Frontal cortex (*n* = 18)	1.4 ± 2.3	0.02 ± 0.03	219 ± 442	1.17 ± 0.44	0.03 ± 0.06
Hippocampus (*n* = 15)	3.8 ± 11.8	0.08 ± 0.25	595 ± 1809	1.24 ± 0.54	0.13 ± 0.38
Red nucleus (*n* = 18)	1.0 ± 1.3	0.01 ± 0.02	140 ± 236	1.57 ± 0.21	0.02 ± 0.03
Substantia nigra (*n* = 17)	1.5 ± 2.8	0.02 ± 0.05	183 ± 460	1.39 ± 0.41	0.03 ± 0.09
	*p*-value	*p*-value	*p*-value	*p*-value	*p*-value
Differences by section	0.91	0.76	0.59	0.003	0.49

**Table 2:** Comparisons of dolphin brain immunohistochemical (IHC) staining of inflammatory indices across four sections in bottlenose dolphins (*Tursiops truncatus*) (*n* = 19). Wilcoxon rank sums test was used for comparisons among brain sections. Significance was defined as a *p* value ≤ 0.05.

IHC Staining by Brain Section	CD68^+^ (Cells per mm^2^)	Mean Stain Intensity			
		IFN-γ	IL-6	TGF-β	TNFα
	mean ± SD	mean ± SD	mean ± SD	mean ± SD	mean ± SD
Frontal cortex (*n* = 18)	0.34 ± 0.45	5.6 ± 2.5	5.0 ± 2.3	6.3 ± 2.9	22 ± 9
Hippocampus (*n* = 15)	0.46 ± 0.38	5.8 ± 2.8	4.9 ± 3.2	6.8 ± 3.1	21 ± 12
Red nucleus (*n* = 18)	0.18 ± 0.24	4.4 ± 1.7	3.4 ± 1.7	6.9 ± 2.1	18 ± 10
Substantia nigra (*n* = 17)	0.12 ± 0.14	4.2 ± 1.9	3.6 ± 2.2	5.4 ± 3.1	16 ± 9
	*p*-value	*p*-value	*p*-value	*p*-value	*p*-value
Differences by section	0.002	0.198	0.168	0.291	0.206

**Table 3:** Antagonist activities of C15:0 across a variety of mechanistic targets included in the Alzheimer’s MoA LeadHunter Panel from Eurofins Discovery *.

Assay Name	Antagonist Response (Average % Inhibition Compared to Positive Control)	Met Antagonist Criteria (Where ≥50% Inhibition = Y, <50% Inhibition = N)
Cholinesterase, Acetyl, ACES	44.7	N
Cholinesterase, Butyryl, CHLE	2.5	N
Peptidase, CTSB (Cathepsin B)	−35.2	N
Cyclooxygenase COX-1	−17.0	N
Cyclooxygenase COX-2	5.3	N
Myeloperoxidase	44.8	N
IDO1	39.4	N
Monoamine Oxidase MAO-A	11.4	N
Monoamine Oxidase MAO-B	64.7	Y
Peptidase, Endothelin Converting Enzyme-1 (ECE-1)	7.1	N
Peptidase, Metalloproteinase, Neutral Endopeptidase	2.1	N
Peptidase, BACE1 (beta-Secretase)	2.4	N
Protein Tyrosine Kinase, Fyn	−16.2	N
Protein Serine/Threonine Kinase, GSK3B	15.0	N
Protein Serine/Threonine Kinase, MAPK14 (p38alpha)	−14.8	N
Protein Tyrosine Phosphatase, DUSP22	−28.1	N
Fatty Acid Amide Hydrolase (FAAH)	70.3	Y
Nitric Oxide Synthetase, Inducible (iNOS)	9.6	N
Catechol-O-Methyl Transferase (COMT)	1.5	N
Lipoxygenase 15-LOX-2	8.0	N
Adenosine A2A	10.7	N
Transporter, GABA	−3.3	N
GABAA, Ro-15-1788, Hippocampus	−16.4	N
Glutamate, AMPA	−1.7	N
Glutamate, NMDA, MK-801	−2.2	N
Glutamate, NMDA, Polyamine	0.4	N
Glutamate, Metabotropic, mGlu5	0	N
Histamine H3	1.7	N
Chemokine CX3CR1	5.3	N
Muscarinic M1	9.0	N
Nicotinic Acetylcholine alpha7, Methyllycaconitine	−9.1	N
Serotonin (5-Hydroxytryptamine) 5-HT1A, WAY-100635	3.9	N
Serotonin (5-Hydroxytryptamine) 5-HT4B	−1.7	N
Serotonin (5-Hydroxytryptamine) 5-HT6	−16.1	N
Sodium Channel, Site 1	−1.3	N
Somatostatin sst4	−4.4	N
Sigma 2	6.6	N
Sigma1	−18.6	N
Platelet Activating Factor (PAF)	−6.6	N

* Data as reported by Eurofins Discovery as part of its Alzheimer’s MoA LeadHunter Panel using standardized enzyme and radioligand binding assays. These data are based on 10 ascending concentrations (up to 50 µM) of pure, free fatty acid C15:0.

**Table 4:** Summary of immunohistochemical (IHC) staining conditions for each antibody.

Antibody	Supplier and Catalog	Lot	Host	Dilution	Antigen Retrieval
Beta amyloid	Cell Signaling (Danvers, MA, USA) #2450	3	Mouse	1:200	ER2(20)
IL-6	Abcam (Cambridge, UK) #ab6672	GR309805-5	Rabbit	1:100	ER1(20)
IFN-γ	Kingfisher (Saint Paul, MN, USA) #PB0494-200	DP1882KM	Goat	1:1600	ER1(20)
TNF-α	Abcam #ab1793	GR32063-1	Mouse	1:100	ER1(20)
TGF-β1	Abcam #ab92486	GR175622-2	Rabbit	1:250	ER2(20)
CD68	Thermo Fisher (Carlsbad, CA, USA) #MA5-13324	SH2440231	Mouse	1:100	ER1(20)

## Data Availability

Full data are provided as a supplement in this manuscript (Table S1).

## Supplementary Materials

The supporting information can be downloaded at: https://www.mdpi.com/article/10.3390/ijms26083746/s1.

## Abbreviations

The following abbreviations are used in this manuscript:

AD	Alzheimer’s disease
Aβ	Amyloid beta
C15:0	Pentadecanoic acid
FAAH	Fatty acid amide hydrolase
MAO-B	Monoamine oxidase B
CD68+	Cluster of differentiation 68
MMP	U.S. Navy Marine Mammal Program
OCFA	Odd-chain saturated fatty acid
AMPK	AMP-activated protein kinase
AKT	PI3K-AKT signaling pathway
PPARα/δ	Peroxisome proliferator-activated receptors alpha/delta
mTOR	Mammalian target of rapamycin
JAK-STAT	Janus kinase—Signal transducer and activator of transcription
HDAC-6	Histone deacetylase 6
LDL	Low density lipoprotein
ALT	Alanine aminotransferase
AST	Aspartate aminotransferase
FFPE	Formalin-fixed paraffin-embedded
IHC	Immunohistochemical
DAB	3, 3 -diaminobenzidine
IL-6	Interleukin-6
IFN-γ	Interferon-gamma
TNF-α	Tumor necrosis factor-alpha
TGF-β1	Transforming growth factor-beta 1
MoA	Mechanism of action
ROS	Reactive oxygen species
DIOS	Dysmetabolic iron overload syndrome
MASLD	Metabolic dysfunction-associated steatotic liver disease
MASH	Metabolic dysfunction-associated steatohepatitis
CB1	Cannabinoid receptor 1
CB2	Cannabinoid receptor 2
ECS	Endocannabinoid system
PD	Parkinson’s disease

## References

[1] A Blueprint for Dementia Research. https://www.who.int/publications/i/item/9789240058248.

[2] Zhang J., Zhang Y., Wang J., Xia Y., Zhang J., Chen L. (2024). Recent advances in Alzheimer’s disease: Mechanisms, clinical trials and new drug development strategies. Signal Trans. Target. Ther..

[3] Muller M., Tang M.X., Schupf N., Manly J.J., Mayeux R., Luchsinger J.A. (2007). Metabolic syndrome and dementia risk in a multiethnic elderly cohort. Dement. Geriatr. Cogn. Disord..

[4] Yaffe K., Kanaya A., Lindquist K., Simonsick E.M., Harris T., Shorr R.I., Tylavsky F.A., Newman A.B. (2004). The metabolic syndrome, inflammation, and risk of cognitive decline. JAVMA.

[5] Raffaitin C., Gin H.G., Empana J.P., Helmer C., Berr C., Tzourio C., Portet F., Dartigues J.F., Alperovitch A., Barberger-Gateau P. (2008). Metabolic syndrome and risk for incident Alzheimer’s disease or vascular dementia. Diabetes Care.

[6] Holmes C., Cairns N., Lantos P., Mann A. (1999). Validity of current clinical criteria for Alzheimer’s disease, vascular dementia, and dementia with Lewy bodies. Br. J. Psychiatr..

[7] Mohammadi S., Ghaderi S., Fatehi F. (2024). Iron accumulation/overload and Alzheimer’s disease risk factors in the precuneus region: A comprehensive narrative review. Aging Med..

[8] Lanctot K.L., Hahn-Pedersen J.H., Eichinger C.S., Freeman C., Clark A., Tazazona L.R.S., Cummings J. (2024). Burden of illness in people with Alzheimer’s disease: A systematic review of epidemiology, comorbidities, and mortality. J. Prev. Alzheimer’s Dis..

[9] Nie Y., Chu C., Qin Q., Shen H., Wen L., Tang Y., Qu M. (2023). Lipid metabolism and oxidative stress in patients with Alzheimer’s disease and amnestic mild cognitive impairment. Brain Pathol..

[10] Zhao H.B., Yang Y. (2024). Hearing loss promotes Alzheimer’s disease. Nat. Aging.

[11] Venn-Watson S., Jensen E.D., Smith C.R., Xitco M., Ridgway S.H. (2015). Annual survival, mortality, and longevity of bottlenose dolphins (*Tursiops truncatus*) at the U.S. Navy Marine Mammal Program, 2004–2013. J. Am. Vet. Med. Assoc..

[12] Venn-Watson S., Smith C.R., Gomez F., Jensen E.D. (2011). Physiology of aging among healthy, older bottlenose dolphins (*Tursiops truncatus*): Comparisons with aging humans. J. Comp. Phys. B.

[13] Venn-Watson S., Smith C., Stevenson S., Parry C., Daniels R., Jensen E., Cendejas V., Balmer B., Janech M., Neely B.A. (2013). Blood-based indicators of insulin resistance and metabolic syndrome in bottlenose dolphins (*Tursiops truncatus*). Front. Endocrinol..

[14] Mazzaro L.M., Johnson S.P., Fair P.A., Bossart G., Carlin K.P., Jensen E.D., Smith C.R., Andrews G.A., Chavey P.S., Venn-Watson S. (2012). Iron indices among bottlenose dolphins (*Tursiops truncatus*): Identifying populations at risk for iron overload. Comp. Med..

[15] Venn-Watson S., Benham C., Carlin K., Leger J.S. (2012). Hemochromatosis and fatty change: Building evidence for insulin resistance in bottlenose dolphins (*Tursiops truncatus*). J. Zoo Wildl. Med..

[16] Venn-Watson S., Carlin K., Ridgway S. (2011). Dolphins as animal models for type 2 diabetes: Sustained, postprandial hyperglycemia and hyperinsulinemia. Gen. Comp. Endocrinol..

[17] Venn-Watson S., Parry C., Baird M., Stevenson S., Carlin K., Daniels R., Smith C.R., Jones R., Wells R.S., Ridgway S. (2015). Increased dietary intake of saturated fatty acid heptadecanoic acid (C17:0) associated with decreasing ferritin and alleviated metabolic syndrome in dolphins. PLoS ONE.

[18] Venn-Watson S., Baird M., Novick B., Parry C., Jensen E.D. (2020). Modified fish diet shifted serum metabolome and alleviated chronic anemia in bottlenose dolphins (*Tursiops truncatus*): Potential role of odd-chain saturated fatty acids. PLoS ONE.

[19] Venn-Watson S., Lumpkin R., Dennis E.A. (2020). Efficacy of dietary odd-chain saturated fatty acid pentadecanoic acid parallels broad associated health benefits in humans: Could it be essential?. Sci. Rep..

[20] Dornan K., Gunenc A., Oomah B.D., Hosseinian F. (2021). Odd chain fatty acids and odd chain phenolic lipids (alkylresorcinols) are essential for diet. J. Am. Chem. Soc..

[21] Ciesielski V., Guerbette T., Fret L., Succar M., Launay Y., Dahirel P., Legrand P., Vlach M., Blat S., Rioux V. (2025). Dietary pentadecanoic acid supplementation at weaning in essential fatty acid-deficient rats shed light on the new family of odd-chain n-8 PUFAs. J. Nutr. Biochem..

[22] Ciesielski V., Legrand P., Blat S., Rioux V. (2024). New insights on pentadecanoic acid with special focus on its controversial essentiality: A mini-review. Biochimie.

[23] Ruan M., Xu F., Li N., Yu J., Teng F., Tang J., Huang C., Zhu H. (2024). Free long-chain fatty acids trigger early postembryonic development in starved *Caenorhabditis elegans* by suppressing mTORC1. PLoS Biol..

[24] Fu W.C., Li H.Y., Li T.T., Yang K., Chen J.X., Want S.J., Liu C.H., Zhang W. (2021). Pentadecanoic acid promotes basal and insulin-stimulated glucose uptake in C2C12 myotubes. Food Nutr. Res..

[25] Bishop C.A., Machate T., Henkel J., Schulze M.B., Klaus S., Piepelow K. (2023). Hepatadecanoic acid is not a key mediator in the prevention of diet-induced hepatic steatosis and insulin resistance in mice. Nutrients.

[26] To N.B., Truong V.N.P., Ediriweera M.K., Cho S.K. (2022). Effects of combined pentadecanoic acid and tamoxifen treatment on tamoxifen resistance in MCF-7/SC breast cancer cells. Int. J. Mol. Sci..

[27] To N.B., Nguyen Y.T., Moon J.Y., Ediriweera M.K., Cho S.K., To N.B., Nguyen Y.T., Moon J.Y., Ediriweera M.K., Cho S.K. (2020). Pentadecanoic acid, an odd-chain fatty acid, suppresses the stemness of MCF-7/SC human breast cancer stem-like cells through JAK2/STAT3 signaling. Nutrients.

[28] Ediriweera M.K., To N.B., Lim Y., Cho S.K. (2021). Odd-chain fatty acids as novel histone deacetylase 6 (HDAC6) inhibitors. Biochimie.

[29] Venn-Watson S., Schork N. (2023). Pentadecanoic acid (C15:0), an essential fatty acid, shares clinically relevant cell-based activities with leading longevity-enhancing compounds. Nutrients.

[30] Venn-Watson S.K., Butterworth C.N. (2022). Broader and safer clinically-relevant activities of pentadecanoic acid compared to omega-3: Evaluation of an emerging essential fatty acid across twelve primary human cell-based disease systems. PLoS ONE.

[31] Wei W., Wong C.C., Jia Z., Liu W., Liu C., Ji F., Pan Y., Wang F., Wang G., Zhao L. (2023). *Parabacteroides distasonis* uses dietary inulin to suppress NASH via its metabolite pentadecanoic acid. Nat. Microbiol..

[32] Galdiero E., Ricciardelli A., D’Angelo C., de Alteriis E., Maione A., Albarano L., Casillo A., Corsaro M.M., Tutino M.L., Parrillii E. (2021). Pentadecanoic acid against *Candida albicans-Klebsiella pneunmoniae* biofilm: Towards the development of an anti-biofilm coating to prevent polymicrobial infections. Res. Microbiol..

[33] Ricciardelli A., Casillo A., Corsaro M.M., Tutino M.L., Parrilli E., Van Der Mei H.C. (2020). Pentadecanal and pentadecanoic acid coatings reduce biofilm formation of *Staphylococcus epidermidis* on PDMS. Pathog. Dis..

[34] Li Y., Liu Y., Chen Y., Wang K., Luan Y. (2022). Design, synthesis and antitumor activity study of a gemcitabine prodrug conjugated with a HDAC6 inhibitor. Bioorg. Med. Chem. Lett..

[35] Wu Y., Zhang X., Liu X., Zhao Z., Tao S., Xu Q., Zhao J., Dai Z., Zhang G., Han D. (2024). Galactooligosaccharides and *Limosilactobacillus reuteri* synergistically alleviate gut inflammation and barrier dysfunction by enriching *Bacteroides acidifaciens* for pentadecanoic acid biosynthesis. Nat. Commun..

[36] Robinson M.K., Lee E., Ugalde-Nicalo P.A., Skonieczny J.W., Chun L.F., Newton K.P., Schwimmer J.B. (2024). Pentadecanoic acid supplementation in young adults with overweight and obesity: A randomized controlled trial. J. Nutr..

[37] Chooi Y.C., Zhang Q.A., Magkos F., Ng M., Michael N., Wu X., Volchanskaya V.S.B., Lai X., Wanjaya E.R., Elejalde U. (2024). Effect of an Asian-adapted Mediterranean diet and pentadecanoic acid on fatty liver disease: The TANGO randomized controlled trial. Am. J. Clin. Nutr..

[38] Shen J., Yu H., Li K., Ding B., Xiao R., Ma W. (2022). The associations between plasma fatty acid and cognitive function mediated by inflammation in patients with type 2 diabetes melitus. Diabetes Metab. Syndr. Obes..

[39] Brydges C.R., Bhattacharyya S., Dehkordi S.M., Milaneschi Y., Penninx B., Jansen R., Kristal B.S., Han X., Arnold M., Kastenmuller G. (2022). Metabolomic and inflammatory signatures of symptom dimensions in major depression. Brain Behav. Immun..

[40] Gunn-Moore D., Kaidanovich-Beilin O., Iradi M.C.G., Gunn-Moore F., Lovestone S. (2018). Alzheimer’s disease in humans and other animals: A consequence of postreproductive life span and longevity rather than aging. Alzheimer’s Dement..

[41] Di Guardo G. (2018). Alzheimer’s disease, cellular prion protein, and dolphins. Alzheimer’s Dement..

[42] Sacchini S., Diáz-Delgado J., De Los Monteros A.E., Paz Y., De Quirós Y.B., Sierra E., Arbelo M., Herraez P., Fernandez A. (2020). Amyloid-beta peptide and phosphorylated tau in the frontopolar cerebral cortex and in the cerebellum of toothed whales: Aging versus hypoxia. Biol. Open.

[43] Vacher M.C., Durrant C.S., Rose J., Hall A.J., Spires-Jones T.L., Gunn-Moore F., Dagleish M.P. (2023). Alzheimer’s disease-like neuropathology in three species of oceanic dolphin. Eur. J. Neurosci..

[44] Garamszegi S.P., Brzostowicki D.J., Coyne T.M., Vontell R.T., Davis D.A. (2024). TDP-43 and Alzheimer’s disease pathology in the brain of a harbor porpoise exposed to the cyanobacterial toxin BMAA. Toxins.

[45] Orekhova K., Testori C., Giorda F., Grattarola C., Mattioda V., Di Guardo G., Corona C., Castagnaro C., Favole A. (2024). Amyloid-β and phosphorylated tau screening in bottlenose dolphin (*Tursiops truncatus*) and striped dolphin *(Stenella coeruleoalba*) brains from Italy reveals distinct immunohistochemical patterns correlating with age and co-morbidity. PLoS ONE.

[46] Perez S.E., Raghanti M.A., Hof P.R., Kramer L., Ikonomovic M.D., Lacor P.N., Eriwin J.M., Sherwood C.C., Mufson E.J. (2013). Alzheimer’s disease pathology in the neocortex and hippocampus of the western lowland gorilla (Gorilla gorilla gorilla). J. Comp. Neurol..

[47] Rao Y.L., Ganaraja B., Murliamanju B.V., Joy T., Krishnamurthy A., Agrawal A. (2022). Hippocampus and its involvement in Alzheimer’s disease: A review. 3 Biotech.

[48] Bouras C., Hof P.R., Giannakopoulos P., Michel J.P., Morrison J.H. (1994). Regional distribution of neurofibrillary tangles and senile plaques in the cerebral cortex of elderly patients: A quantitative evaluation of a one-year autopsy population from a geriatric hospital. Cereb. Cortex.

[49] Buckner R.L. (2010). The role of the hippocampus in prediction and imagination. Ann. Rev. Psychol..

[50] Lucena P.B., Heneka M.T. (2024). Inflammatory aspects of Alzheimer’s disease. Acta Neuropathol..

[51] Venn-Watson S., Jensen E.D., Schork N.J. (2020). A 25-y longitudinal dolphin cohort supports that long-lived individuals in same environment exhibit variation in aging rates. Proc. Natl. Acad. Sci. USA.

[52] Venn-Watson S. (2024). The Cellular Stability Hypothesis: Evidence of ferroptosis and accelerated aging-associated diseases as newly identified nutritional pentadecanoic acid (C15:0) deficiency syndrome. Metabolites.

[53] Dixon S.J., Lemberg K.M., Lamprecht M.R., Skouta R., Zaitsev E.M., Gleason C.E., Patel D.N., Bauer A.J., Cantley A.M., Yang W.S. (2012). Ferroptosis: An iron-dependent form of nonapoptotic cell death. Cell.

[54] Feng L., Sun J., Xia L., Shi Q., Hou Y., Zhang L., Li M., Fan C., Sun B. (2024). Ferroptosis mechanisms and Alzheimer’s disease. Neural Regen. Res..

[55] Soni P., Kaidery N.A., Sharma S.M., Gazaryan I., Nikulin S.V., Hushpulian D.M., Thomas B. (2024). A critical appraisal of ferroptosis in Alzheimer’s and Parkinson’s disease: New insights into emerging mechanisms and therapeutic targets. Front. Pharmacol..

[56] Majernikova N., Marmolejo-Garza A., Salinas C.S., Luu M.D.A., Zhang Y., Trombetta-Lima M., Tomin T., Birner-Gruenberger R., Lehtonen S., Koistinaho J. (2024). The link between amyloid β and ferroptosis pathway in Alzheimer’s disease progression. Cell Death Dis..

[57] Streit W.J., Phan L., Bechmann I. (2025). Ferroptosis and pathogenesis of neuritic plaques in Alzheimer disease. Pharmacol. Rev..

[58] Egmond N.V., Straub V.M., van der Stelt M. (2021). Targeting endocannabinoid signaling: FAAH and MAG lipase inhibitors. Ann. Rev. Pharmacol. Toxicol..

[59] Lowe H., Toyang N., Steele B., Bryant J., Ngwa W. (2021). The endocannabinoid system: A potential target for the treatment of various diseases. Int. J. Mol. Sci..

[60] Jain S., Sharma S., Paliwal A., Dwivedi J., Paliwal S., Paliwal V., Paliwal S., Sharma J. (2024). Discovery of novel fatty acid amide hydrolase (FAAH) inhibitors as anti-Alzheimer’s agents through pharmacophore-based virtual screening, molecule docking and experimental validation. Med. Chem. Res..

[61] Armeli F., Coccurello R., Giacovazzo G., Mengoni B., Paoletti I., Oddi S., Maccorrone M., Businaro R. (2024). FAAH inhibition counteracts neuroinflammation via autophagy recovery in AD models. Int. J. Mol. Sci..

[62] Rong K., Li Z., Wu X., Gao S., Zhao J., Yang J., Jiang X., Zhang J., Tang W. (2025). Natural phenol carbamates: Selective BuChE/FAAH dual inhibitors show neuroprotection in an Alzheimer’s disease mouse model. Eur. J. Med. Chem..

[63] Brunetti L., Laghezza A., Fulvio L., Paolo T., Luca P. (2020). Combining fatty acid amide hydrolase (FAAH) inhibition with peroxisome proliferator-activated receptor (PPAR) activation: A new potential multi-target therapeutic strategy for the treatment of Alzheimer’s disease. Neural Regen. Res..

[64] Jian S., Bisht A., Verma K., Negi S., Paliwal S., Sharma S. (2021). The role of fatty acid amide hydrolase enzyme inhibitors in Alzheimer’s disease. Cell Biochem. Funct..

[65] Roy R.G., Mandal P.K., Maroon J.C. (2023). Oxidative Stress Occurs Prior to Amyloid Aβ Plaque Formation and Tau Phosphorylation in Alzheimer’s Disease: Role of Glutathione and Metal Ions. ACS Chem. Neurosci..

[66] Chen L.L., Fan Y.G., Zhao L.X., Zhang Q., Wang Z.Y. (2023). The metal ion hypothesis of Alzheimer’s disease and the anti-neuroinflammatory effect of metal chelators. Bioorg. Chem..

[67] Steen E., Terry B.M., Rivera E.J., Cannon J.L., Neely T.R., Tavares R., Xu X.J., Wands J.R., de la Monte S.M. (2005). Impaired insulin and insulin-like growth factor expression and signaling mechanisms in Alzheimer’s disease–is this type 3 diabetes?. J. Alzheimers Dis..

[68] Gabbouj S., Ryhanen S., Marttinen M., Wittrahm R., Takalo M., Kemppainen S., Martiskainen H., Tanila H., Happasalo A., Hiltunen M. (2019). Altered insulin signaling in Alzheimer’s disease brain—Special emphasis on PI3K-Akt pathway. Front. Neurosci..

[69] Querfurth H., Lee H.K. (2021). Mammalian/mechanistic target of rapamycin (mTOR) complexes in neurodegeneration. Mol. Neurodegen..

[70] Zhao H., Liu Y., Cai N., Liao X., Tang L., Wang Y. (2024). Endocannabinoid hydrolase inhibitors: Potential novel anxiolytic drugs. Drug Des. Dev. Ther..

[71] Portugalov A., Akirav I. (2024). FAAH inhibition reverses depressive-like behavior and sex-specific neuroinflammatory alterations induced by early life stress. Cells.

[72] Barbetti M., Mancabelli L., Vacondio F., Langhi G., Ferlenghi F., Viglioli M., Turroni F., Carnevali L., Mor M., Ventura M. (2024). Social stress-induced depressive-like symptoms and changes in gut microbial and lipidomic profiles are prevented by pharmacological inhibition of FAAH activity in male rats. Prog. Neuro Psychopharmacol. Biol. Psychiatry.

[73] Schmidt M.E., Liegowitz M.R., Stein M.B., Grunfeld J., Van Hove I., Simmons W.K., Van Der Ark P., Palmer J.A., Saad Z.S., Pemberton D.J. (2021). The effects of inhibition of fatty acid amide hydrolase (FAAH) by JNJ-42165279 in social anxiety disorder: A double-blind, randomized, placebo-controlled proof-of-concept study. Neuropsychopharmacology.

[74] Venn-Watson S., Reiner J., Jensen E.D. (2022). Pentadecanoylcarnitine is a newly discovered endocannabinoid with pleiotropic activities relevant to supporting physical and mental health. Sci. Rep..

[75] Zanfirescu A., Nitulescu G., Mihai D.P., Nitulescu G.M. (2022). Identifying FAAH inhibitors as new therapeutic options for the treatment of chronic pain through drug repurposing. Pharmaceut.

[76] Larauche M., Mulak A., Ha C., Million M., Arnett S., Germano P., Pearson J.P., Currie M.G., Tache Y. (2024). FAAH inhibitor URB597 shows anti-hyperalgestic action and increases brain and intestinal tissues fatty acid amides in a model of CRF_1_ agonist mediated visceral hypersensitivity in male rats. Neurogastroenterol. Motil..

[77] Chen C., Wang W., Poklis J.L., Li P.L., Lichtman A.H., Gewirtz D.A., Li N. (2025). Mitigation of cisplatin-induced acute kidney injury through oral administration of fatty acid amide hydrolase inhibitor PF-04457845. J. Pharmacol. Exp. Ther..

[78] Knoll J. (1983). Deprenyl (selegiline): The history of its development and pharmacological action. Acta Neurol. Scan. Suppl..

[79] Wang Y., Wang Z. (2024). Effects and safety of monoamine oxidase-B inhibitors for early Parkinson’s Disease: A network meta-analysis. Eur. Neurol..

[80] Oyovwi M.O., Udi O.A., Atere A.D., Joseph G.U., Ogbutor U.G. (2025). Molecular pathways: The quest for effective MAO-B inhibitors in neurodegenerative therapy. Mol. Biol. Rep..

[81] Schedin-Weiss S., Inoue M., Hromadkova L., Teranishi Y., Yamamoto N.G., Weihager B., Bogdanovic N., Winblad B., Sandebring-Matton A., Frykman S. (2017). Monoamine oxidase B is elevated in Alzheimer disease neurons, is associated with ɣ-secretase and regulates neuronal amyloid ꞵ-peptide levels. Alzheimer’s Res. Ther..

[82] Weinreb O., Amit T., Bar-Am O., Youdim M.B.H. (2015). Neuroprotective effects of multifaceted hybrid agents targeting MAO, cholinesterase, iron and β-amyloid in again and Alzheimer’s disease. Br. J. Pharmacol..

[83] Thomas T. (2000). Monoamine oxidase-B inhibitors in the treatment of Alzheimer’s disease. Neurobiol. Aging.

[84] Mohamadpour B., Mirazi N., Komaki A., Basir H.S., Hosseini A. (2024). Protective effects of selegiline against amyloid beta-induced anxiety-like behavior and memory impairment. Brain Behav..

[85] Rullo M., La Spada G., Catto M., Pisani L. (2024). Monoamine oxidase inhibition for the treatment of neurodegenerative diseases: Rationale, assay methodologies, and reference compounds. Methods Neurodegener. Dis. Drug Discov..

[86] Naoi M., Maruyama W. (2010). Monoamine oxidase inhibitors as neuroprotective agents in age-dependent neurodegenerative disorders. Curr. Pharm. Des..

[87] Sekar P.V.C., Thomas S.M., Veerabathirn R. (2024). An overview of the role of monoamine oxidase-B in Parkinson’s disease: Implications for neurodegeneration and therapy. Explor. Neuroprot. Ther..

[88] Alborghetti M., Bianchini E., De Carolis L., Galli S., Pontieri F.E., Domiziana R. (2024). Type-B monoamine oxidase inhibitors in neurological diseases: Clinical applications based on preclinical findings. Neural Regen. Res..

[89] Naio M., Maruyama W., Shamoto-Nagai M. (2018). Type A and B monoamine oxidases distinctly modulate signal transduction pathway and gene expression to regulate brain function and survival of neurons. Neuro Preclin. Neurol. Stud..

[90] Banerjee C., Tripathy D., Kumar D., Chakraborty J. (2024). Monoamine oxidase and neurodegeneration: Mechanisms, inhibitors and natural compounds for therapeutic intervention. Neurochem. Int..

[91] Dormegny-Jeanjen L.C., Mainberger O.A.E., Crespin de Billy C., Obrecht A., Danila V., Erb A., Arcay H.M., Weibel S., Blanc F., Meyer G. (2024). Safety and tolerance of combination of monoamine oxidase inhibitors and direct dopamine agonists in adults and older adults with highly resistant depression. L’enchephale.

[92] Karalija N., Papenberg G., Johansson J., Wahlin A., Salami A., Andersson M., Axelsson J., Kuznetsov D., Riklund K., Lövdén D. (2024). Longitudinal support for the correlative triad among aging, dopamine D2-like receptor loss, and memory decline. Neurobiol. Aging.

[93] Coleman C.R., Pallos J., Arreola-Bustos A., Martin I. (2024). Natural variation in age-related dopamine neuron degeneration is glutathione dependent and linked to life span. Proc. Natl. Acad. Sci. USA.

[94] Ruehl W.W., Enriken T.L., Muggenburg B.A., Bruyette D.S., Griffith W.C., Hahn F.F. (1997). Treatment with L-deprenyl prolongs life in elderly dogs. Life Sci..

[95] Knoll J. (1988). The striatal dopamine dependency of life span in male rats. Longevity study with (−) deprenyl. Mech. Ageing Dev..

[96] Knoll J. (1995). Rationale for (−) deprenyl (selegiline) medication in Parkinson’s disease and in prevention of age-related nigral changes. Biomed. Pharmacol..

